# Hydrogen Evolution-Directed Electrodeposition of a Cobalt Selenide/Cobalt Oxide Electrocatalyst for the Hydrogen and Oxygen Evolution Reactions

**DOI:** 10.1021/acsaem.5c02096

**Published:** 2025-08-06

**Authors:** Gillian Collins, Daniele Alves, Tara Barwa, Raj Karthik, Sukanya Ramaraj, Carmel B. Breslin

**Affiliations:** † Department of Chemistry, 8798 Maynooth University, Maynooth, Co. Kildare W23F2H6, Ireland; ‡ Kathleen Lonsdale Institute, Maynooth University, Maynooth, Co. Kildare W23F2H6, Ireland

**Keywords:** renewable hydrogen, electrodeposition, cobalt oxide, cobalt diselenide, oxygen evolution reaction, hydrogen evolution reaction

## Abstract

As more emphasis is placed on renewable energy worldwide, the electrochemical splitting of water has attracted considerable attention. Herein, a simple and cost-effective electrocatalyst was studied for both the hydrogen and oxygen evolution reactions. The electrocatalyst consisted of an electrodeposited cobalt diselenide/cobalt oxide composite, which was fabricated from an aqueous solution containing cobalt and selenium salts on a glassy carbon substrate. The optimal deposition conditions were found to be a fixed potential at −1.2 V vs Ag/AgCl for 400 s. At this relatively low potential, the electrodeposition process is accompanied by the hydrogen evolution reaction, resulting in a hierarchical, flower-like morphology. The electrochemical active surface area was calculated to be 89 cm^2^ in 1.0 M KOH and 72 cm^2^ in 0.5 M H_2_SO_4_. This electrocatalyst facilitated the hydrogen evolution reaction in both KOH and H_2_SO_4_ solutions, with overpotentials of 305 and 205 mV at 10 mA cm^–2^, respectively. Similarly, good oxygen evolution activity was achieved in KOH. The electrocatalyst demonstrated good stability over a 24 h period, with evidence of improved oxygen evolution activity following the stability studies.

## Graphical abstract

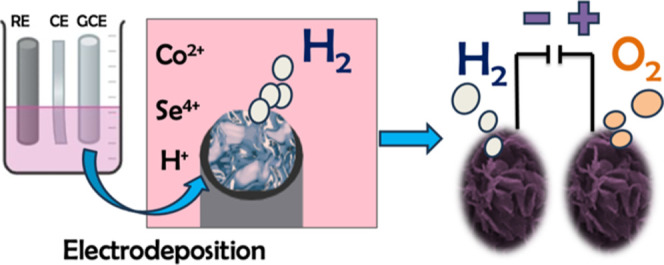

## 1 Introduction

The global transition from fossil fuel dependence to low-carbon energy sources is key for mitigating climate change. Unfortunately, fossil fuels are still the source of most of the world’s energy, leading to pollution of the environment.^1^ Solar, wind, and wave power are renewable sources of energy with the additional benefit of being clean and abundant and are being developed to alleviate the effects of climate change.^2^ Within this renewable group, electrochemical water splitting (water electrolysis) is a sustainable method that utilizes renewable energy sources to split water and generate hydrogen and oxygen.^3,4^ Hydrogen, when produced using renewable options, is a clean and sustainable energy carrier with water as its only byproduct when used as a fuel. However, despite these obvious advantages, the practical application of hydrogen production using electrochemical water splitting is hindered by limitations in electrocatalysts, cost, and stability.

Noble metal-based electrocatalysts, based on Pt, Ru and Ir, remain the most efficient for the splitting of water. However, these are expensive with limited reserves.^5^ More recently, earth-abundant electrocatalysts comprising Fe, Co, and Ni-based sulfides, selenides, and phosphides have attracted considerable attention.^3,5^ Among these systems, transition metal dichalcogenides (TMDs) are of particular interest due to their tunable structures.^3,6,7^ Cobalt diselenide (CoSe_2_) has been shown to be a promising electrocatalyst for water splitting due to its favorable electronic configuration and stability in both acidic and alkaline media.^8^ Recently, CoSe_2_ has also been combined with other electrocatalysts to give hybrids or composites for water splitting. Various composites, including CoSe_2_/CoO core–shell microspheres,^9^ bimetallic NiSe_2_–CoSe_2_ ^10^ and Ni/CoSe_2_ at copper foam supports,^11^ and CoSe_2_ combined with a CoNi layered double hydroxide (LDH)^12^ have all been employed for water splitting. Likewise, composites comprising FeSe_2_/CoSe_2_/NS-doped carbon,^13^ nanothorn-like Nb/CoSe_2_,^14^ NiSe_2_/CoSe_2_ consisting of nanorods and nanoparticles,^15^ and cube-shaped nanostructured CoSe_2_ ^16^ have been utilized for the oxygen evolution reaction (OER). These TMD electrocatalysts are typically synthesized as powders and then coated onto a suitable substrate.^6^ On the other hand, it is possible to electrodeposit TMDs directly onto a substrate, and this has the added advantage that immobilization of the electrocatalyst is not needed, and furthermore, binders are not required.^17^ However, in a recent study, it was found that electrodeposited CoSe exhibited poor stability as a HER electrocatalyst in acidic media, and good stability was only achieved following an additional annealing step at 300 °C,^18^ in agreement with a previous study that employed annealing temperatures between 250 and 600 °C.^19^ Although the electrodeposition period is short, the additional annealing steps at relatively high temperatures make the fabrication of these electrocatalysts more energy-intensive, approaching the energy required in hydrothermal synthesis.

Therefore, in this study, a CoSe_2_ composite was electrodeposited with the aim of achieving good HER activity and stability in both alkaline and acidic media. Both CoSe_2_ and a cobalt oxide (CoO_*x*_) were coelectrodeposited to give a composite from a single aqueous solution containing cobalt and selenium salts on a simple glassy carbon substrate. The electrodeposited electrocatalyst, featuring a unique flower-like hierarchical structure, was achieved in a very short period of 400 s, without the need for annealing, additional binders, or other modifications. The unique flower-like architecture, achieved through the simultaneous electrodeposition and hydrogen evolution, features several thin and extended petals, providing an open network with a high surface area that enables efficient access to both acidic and alkaline electrolytes. This composite was successfully employed as an electrocatalyst for the hydrogen evolution reaction (HER) in both acidic and alkaline solutions, and it also served as an efficient electrocatalyst for the OER in alkaline conditions, all with equally good stability over a period of 24 h.

## 2 Experimental Section

### 2.1 Chemical Reagents

Cobalt­(II) acetate tetrahydrate ((CH_3_COO)_2_Co·4H_2_O), sodium selenite (Na_2_SeO_3_), potassium chloride (KCl), potassium hydroxide (KOH), sulfuric acid (H_2_SO_4_), and hydrochloric acid (HCl) were supplied by Merck. All reagents were analytical grade and used without further purification. All aqueous solutions were prepared using deionized water (DI water).

### 2.2 Electrodeposition Experiments

The electrodeposition experiments were carried out in an aqueous solution containing 20 mM (CH_3_COO)_2_Co·4H_2_O) and 5 mM Na_2_SeO_3_, with 0.1 M KCl serving as the supporting electrolyte. To ensure good solubility of the reagents, the pH was adjusted to 2.3 using a small volume of concentrated hydrochloric acid (HCl). A glassy carbon electrode (GCE, 3 mm diameter) was used as the substrate. Prior to the electrodeposition reactions, the surface of the GCE was polished using a 1 μm diamond particle suspension on a microcloth, thoroughly rinsed in DI water, sonicated for 1 min, and dried under a stream of air. A three-electrode cell, comprising a polished GCE, a high surface area Pt counter electrode and an Ag/AgCl reference, was employed in all electrodeposition experiments. The electrodeposit was performed using a fixed potential of −1.2 V vs Ag/AgCl for 400 s, which was selected following optimization of the electrodeposition potential and time. Following electrodeposition, the deposit was thoroughly rinsed with DI water and designated as EDC@GCE. For comparison purposes, the electrodeposition of cobalt oxide from 20 mM (CH_3_COO)_2_Co·4H_2_O) and 0.1 M KCl, and selenium oxide from 5 mM Na_2_SeO_3_, and 0.1 M KCl were also studied.

### 2.3 Characterization

The electrodeposited composite was characterized using a combination of SEM, XRD, Raman, and XPS techniques. Details on the morphology were provided with a FE-SEM Hitachi S-4800 microscope. Information regarding the crystallinity of the optimized deposit was obtained using a powder X-ray diffraction (P-XRD PANalytical X’Pert-PRO MPD) system. Raman spectra were recorded with a Raman XploRA PLUS Horiba system, employing an excitation wavelength of 532 nm, while the XPS analyses were conducted using a Kratos AXIS ULTRA spectrometer.

### 2.4 Electrochemical Testing

Cyclic voltammetry (CV), linear sweep voltammetry (LSV) and chronoamperometry tests were performed to examine the catalytic activity of the materials. Electrochemical measurements were performed using a Solartron SI-1287 electrochemical workstation with a three-electrode system and a 1.0 M KOH or 0.5 M H_2_SO_4_ electrolyte solution. The modified GCE and a Hg/HgO electrode were employed as the working and reference electrodes, respectively, in alkaline solutions, while an Ag/AgCl electrode was used as the reference electrode in acidic environments. The electrode potentials were converted to the reversible hydrogen electrode (RHE) scale using the relationship, *E*_RHE_ = *E*_Meas_ + *E*_Hg/HgO_ (0.098) + 0.0592 pH with the Hg/HgO reference electrode. For the acidic solutions, the potential was converted using the potential of the Ag/AgCl, *E*_RHE_ = *E*_Meas_ + *E*_Ag/AgCl_ (0.197) + 0.0592 pH. Platinum counter electrodes were used in the electrodeposition reactions and OER studies. As it has been reported that the chemical and electrochemical dissolution of Pt can significantly improve the HER activity of nonprecious electrocatalysts,^20^ graphite rods were employed as the counter electrodes for the HER investigations. LSV was acquired at a scan rate of 5 mV s^–1^. For Tafel analysis, the LSVs were acquired at the slower scan rate of 0.1 mV s^–1^ and the Tafel slope was computed using eq 1, where η is the overpotential, j is the measured current density, *b* is the Tafel slope, and a is a constant containing the exchange current density, *j*_0_.

1 η=a+blog|j|

### 2.5 Purification of KOH

To determine the influence of residual iron ions in commercial KOH (which has a purity of 90%, with water and carbonates as the main impurities) on the OER activity, the 1.0 M KOH electrolyte was purified using a method proposed by Boettcher and co-workers.^21^ Pure Ni­(OH)_2_ was formed, and this was then used to remove the residual iron in the KOH electrolyte. In a typical experiment, 2.0 g of high-purity Ni­(NO_3_)_2_·6H_2_O was dissolved in DI water to promote the precipitation of Ni­(OH)_2_. The formed Ni­(OH)_2_ was recovered by centrifugation and thoroughly washed with DI water. The KOH (50 mL) was combined with the freshly prepared Ni­(OH)_2_, stirred for 10 min, and then allowed to rest for an additional 3 h period. The purified KOH was finally recovered using centrifugation.

## 3 Results and Discussion

### 3.1 Optimization of the Electrodeposition of the Composite

Typical CVs, cycled from 0.25 V to lower applied potentials, where the reduction of the solution ions and the formation of the electrodeposits were seen, are shown in Figure 1a. Three CVs are presented, corresponding to the cycling of the GCE in the Co^2+^, Se^4+^ and in the combined Co^2+^/Se^4+^ electrolytes. For the Co^2+^-containing solution, a single prominent reduction wave is evident at −1.14 V vs Ag/AgCl. At these low applied potentials, the reduction of Co^2+^ is accompanied by the reduction of H^+^ ions at the deposited cobalt to give a looped reduction wave. As the electrode is cycled to higher potentials, the oxidation of Co to Co^2+^ is seen with a peak potential at −0.19 V vs Ag/AgCl. The electrochemical reduction of Se^4+^ becomes evident at approximately −0.92 V vs Ag/AgCl, and the reduction current, although low, continues to increase as the potential is cycled to more negative values. On the other hand, the CVs recorded in the combined Co^2+^ and Se^4+^-containing electrolyte are very different. During the reduction cycle, three reduction waves emerge. Two small, but narrow, reduction waves are seen at −0.53 and −0.65 V vs Ag/AgCl, followed by a larger reduction wave. Upon reversing the cycling, a single oxidation wave is observed with a peak potential at −0.09 V vs Ag/AgCl. The peak at −0.53 V vs Ag/AgCl appears to be related to the reduction of Se^4+^ to elemental Se, eq 2, while the second peak at the lower potential of −0.65 V vs Ag/AgCl is consistent with the reduction of Se to Se^2–^, eq 3. As the reduction of Co^2+^ occurs at a slightly lower potential (evident from the data recorded in the Co^2+^ solution), it appears that this peak is related to the further reduction of Se. However, some reduction of Co^2+^ to Co or cobalt oxides/hydroxides may occur, facilitated by the selenium-modified surface. The formation of CoSe is possible through the coupling of the adsorbed H_2_Se (Se^2–^) with Co^2+^, eq 4, while the formation of CoSe_2_ can occur through the reaction between H_2_Se and Co^2+^, eqs 5 and 6. The larger wave in the vicinity of −1.0 V vs Ag/AgCl was assigned to eq 6, where the reduction of Co^2+^ is facilitated through the simultaneous reduction of Se^4+^ to give Co and Se.^22^

2 $${HSeO}_{3}^{-}+{5H}^{+}+{4e}^{-}\to Se+{3H}_{2}O$$

3 $$Se+{2e}^{-}\to {Se}^{2-}$$

4 $$H_{2}Se+{Co}^{2+}\to CoSe+{2H}^{+}$$

5 $$H_{2}Se+{Co}^{2+}+Se\to {CoSe}_{2}+{2H}^{+}$$

6 $$H_{2}{SeO}_{3}+{Co}^{2+}+{4H}^{+}+{6e}^{-}\to Se+Co+{3H}_{2}O$$

**Figure 1:**
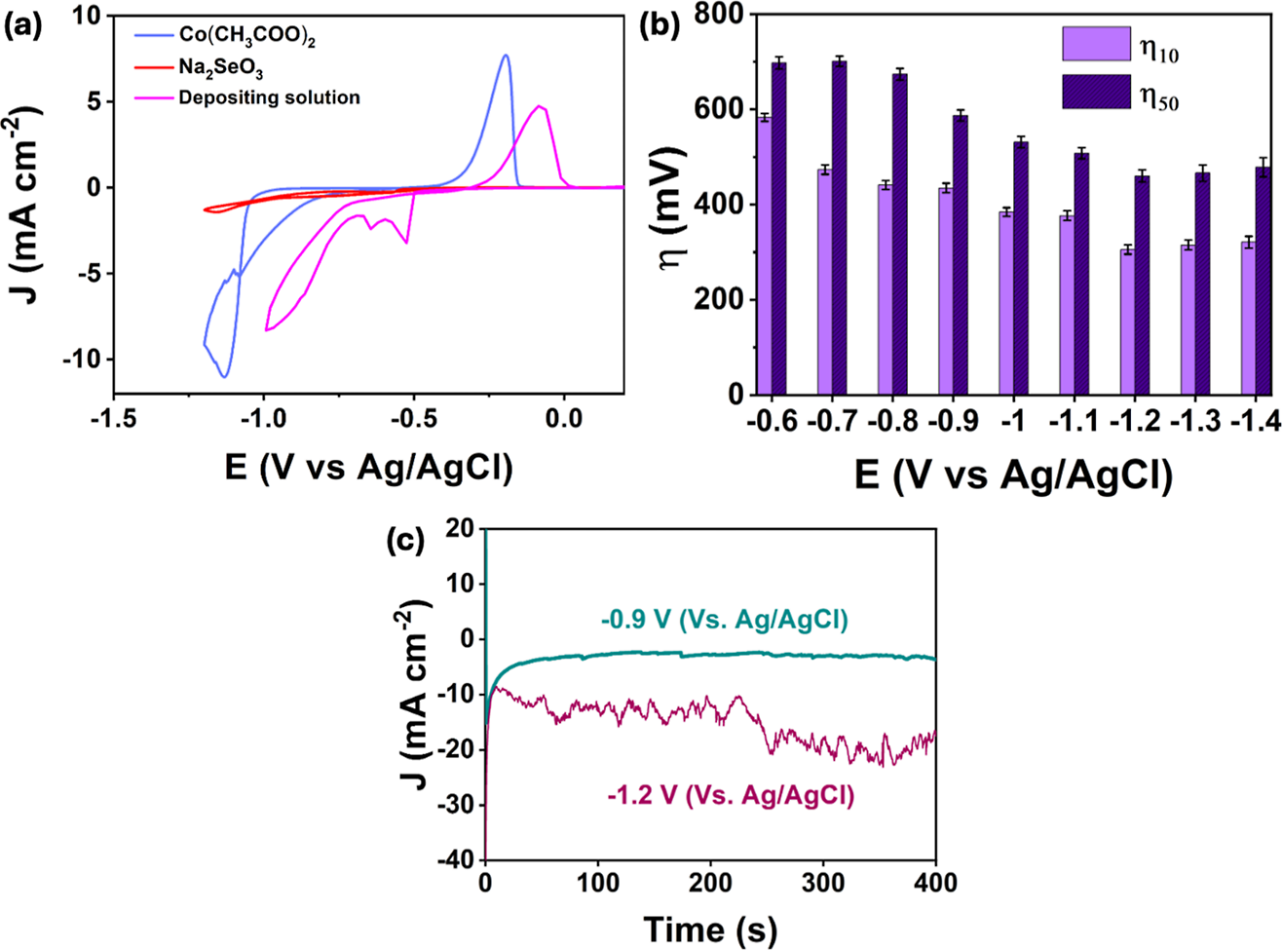
(a) CVs recorded at GCE cycled in 20 mM (CH_3_COO)_2_Co·4H_2_O, 5 mM Na_2_SeO_3_, and the combined solution (20 mM (CH_3_COO)_2_Co·4H_2_O and 5 mM Na_2_SeO_3_) at a pH of 2.3 with 0.1 M KCl from 0.25 to −1.0 or −1.2 V vs Ag/AgCl V at 20 mV s^–1^, (b) overpotentials required to give current densities of 10 and 50 mA cm^–2^ in the HER in 1.0 M KOH, data recorded with a scan rate of 5 mV s^–1^ and (c) current–time plots recorded at −1.2 and −0.9 V vs Ag/AgCl.

The oxidation wave evident at −0.09 V vs Ag/AgCl was assigned to eq 7. However, it may also contain contributions from the oxidation of some electrodeposited Co/Co oxides/hydroxides.

7 $${Co}^{2+}+{2H}_{2}Se\to {CoSe}_{2}+{4H}^{+}+{2e}^{-}$$

It is evident from Figure 1a that the composition of the electrodeposited layers will depend on the applied potentials used in forming the deposit. Therefore, the electrodeposited material was optimized based on its performance for the HER in 1.0 M KOH. A systematic study was conducted at deposition potentials ranging from −0.7 to −1.4 V vs Ag/AgCl for a fixed duration of 400 s. The corresponding data are summarized in Figure 1b, where the overpotentials required to deliver current densities of 10 and 50 mA cm^–2^ for the HER in 1.0 M KOH are presented as a function of the fixed potential used in the deposition of the composite. Among these, a potential of −1.2 V vs Ag/AgCl was identified as the optimal electrodeposition potential, yielding the lowest overpotentials for the HER at both 10 and 50 mA cm^–2^. At −1.2 V vs Ag/AgCl, the applied potential is sufficiently negative to facilitate the deposition of both the cobalt diselenide and cobalt oxides, while enabling the evolution of hydrogen, which in turn can alter the morphology. Much higher overpotentials are observed at applied potentials from −0.6 to −1.1 V, and this appears to be related to the poor electrodeposition of the cobalt oxide/selenide phases. Subsequently, the deposition time was optimized at the fixed potential of −1.2 V (Vs Ag/AgCl). Overpotential measurements at various deposition times revealed that the lowest overpotential occurred at 400 s, as illustrated in Figure S1 (Supporting Information). Thus, 400 s was identified as the optimal deposition time. The current–time plots recorded at −0.9 V and −1.2 V vs Ag/AgCl are shown in Figure 1c. As expected, higher reduction currents are evident at the more negative potential of −1.2 V vs Ag/AgCl, indicating higher rates of electrodeposition. At these lower potentials, the reduction of H^+^ ions and the production of hydrogen bubbles occur, and indeed this is seen as noise in the current signal at −1.2 V vs Ag/AgCl.

### 3.2 Morphological and Structural Analysis of the Composite

The surface morphology of the composite is shown in Figure 2. Flower-like electrodeposits are evident with petal-like sheets extending from the GCE substrate. This somewhat unusual morphology may be related to the low potentials applied during the electrodeposition process. At these potentials, gaseous hydrogen bubbles are produced. Initially, a layer of the electrodeposit is formed, which acts as an electrocatalyst for the HER, as evident in Figure 1c. The evolving *H*_2_(*g*) bubbles influence the growth of the deposit, preventing the formation of a compact structure and contributing to the observed morphology. The growth of the petal-like structures appears to facilitate the removal of the H_2_(*g*) bubbles between the petals. This morphology differs from the typical cauliflower-like deposits observed for electrodeposited CoSe^18^ or the aggregated clusters, with limited surface area, seen for electrodeposited NiCoS_2_ and CoFeSe_2_.^23^ This suggests that the codeposition of cobalt oxides/hydroxides and selenides, coupled with the HER activity during the electrodeposition process, leads to the formation of these hierarchical structures. Indeed, the EDX mapping, as shown in Figure 2, indicates that the electrodeposited flowers consist of Co, Se, and some O, confirming the presence of cobalt oxides/selenide. In addition, the EDX mapping data show that Co, Se, and O are well dispersed across the deposit, resulting in a uniform distribution of the oxide and selenide phases.

**Figure 2:**
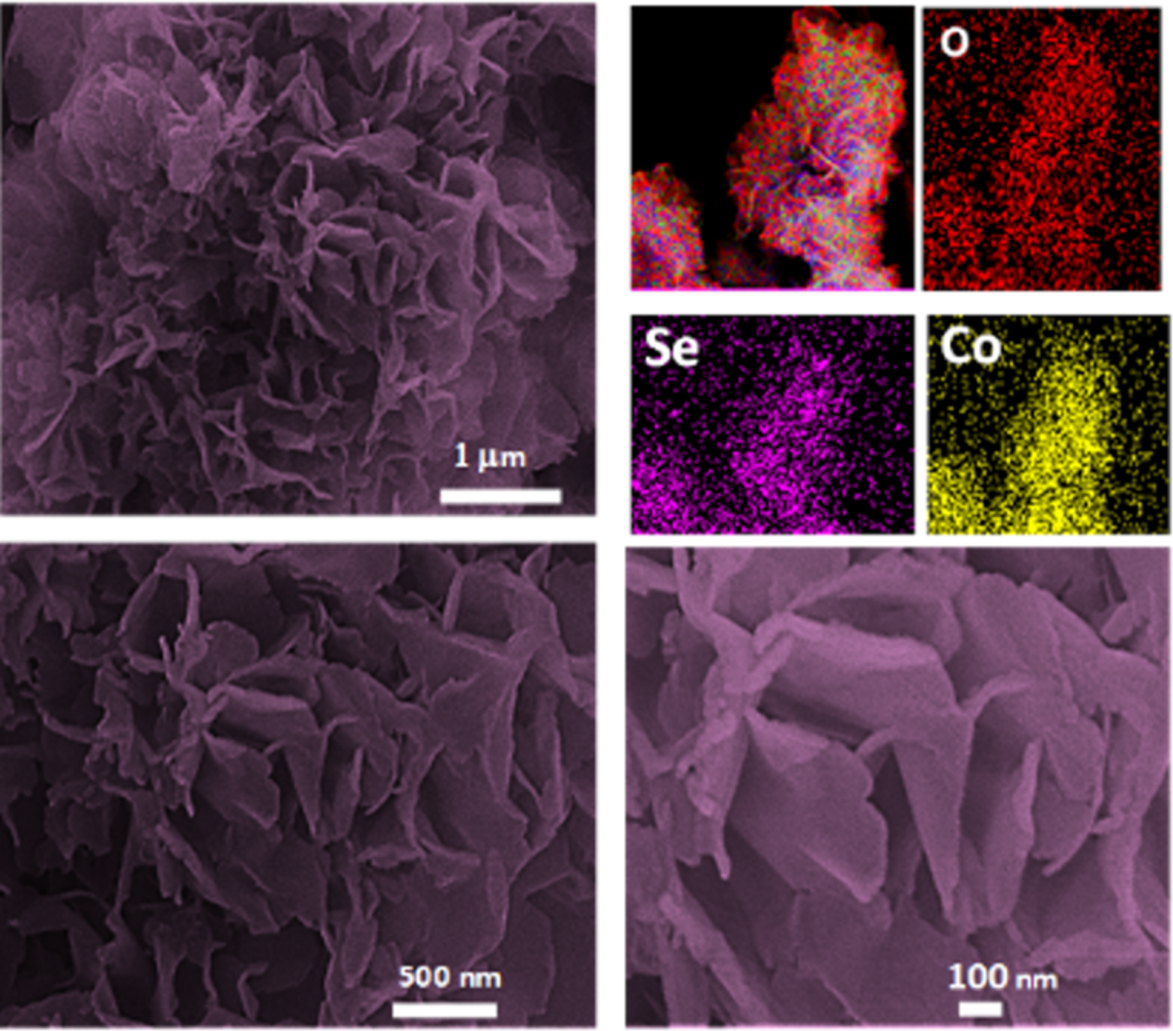
SEM micrographs at different magnifications recorded for the electrodeposited composite and the corresponding EDX mapping for O, Se and Co.

Raman spectroscopy was employed in the analysis of the electrodeposited composite, and the resulting data are shown in Figure 3a. The peak at 185 cm^–1^ is assigned to the Se–Se stretching in CoSe_2_ and corresponds to the A_g_ mode. Likewise, the peaks corresponding to the E_g_, F_2g_^(2)^ and F_2g_^(1)^ and A_1g_ symmetries appear at 465, 501, 603, and 666.1 cm^–1^, respectively, consistent with earlier reports.^24^ The crystallinity of the electrodeposit was probed using XRD, and a typical plot is shown in Figure 3b. Clearly, the electrodeposit exhibits an amorphous-like nature, with no evidence of any prominent diffraction peaks that could be assigned to a cobalt oxide phase.

**Figure 3:**
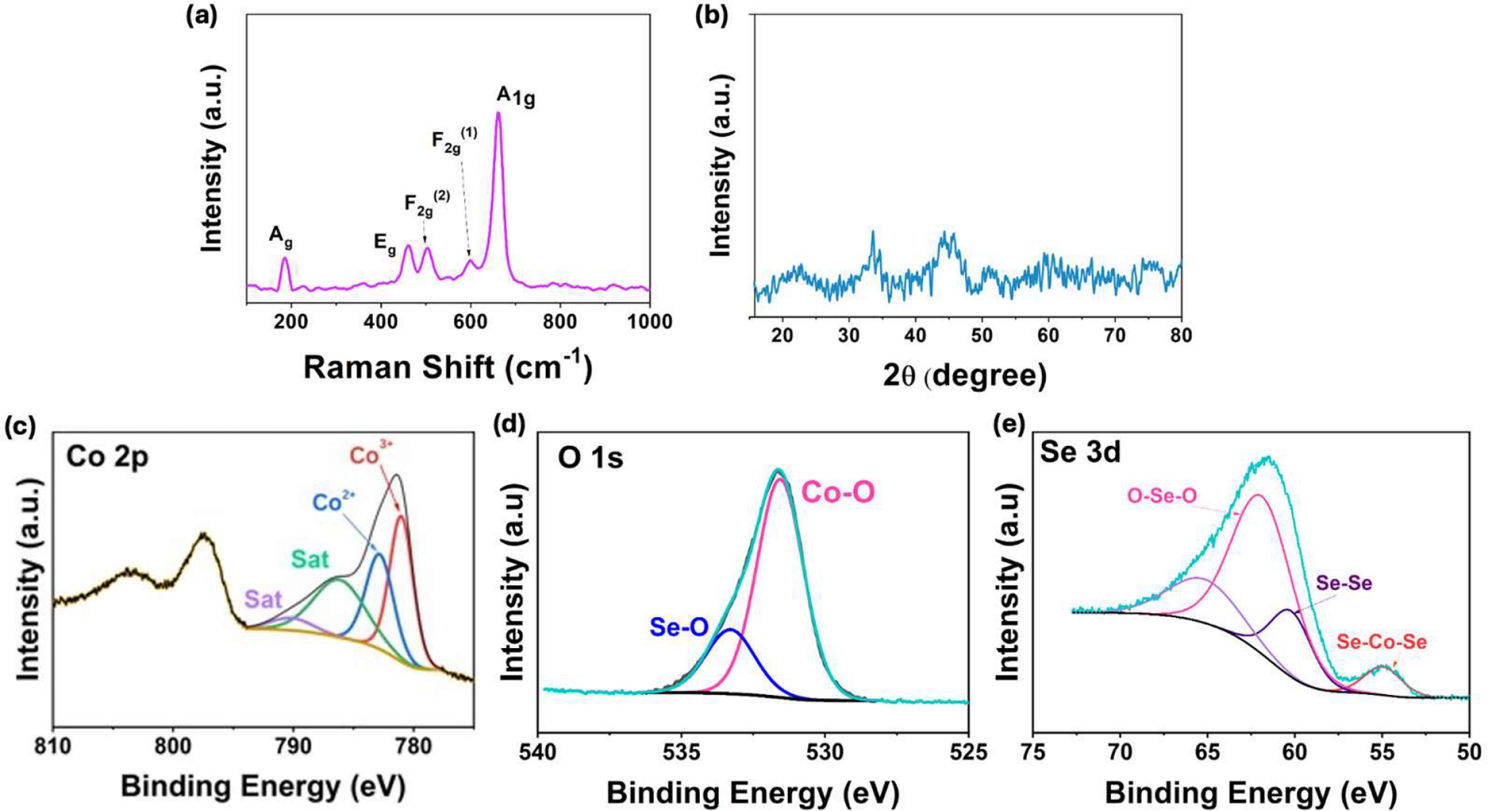
Characterization data for the electrodeposited composite (a) Raman, (b) XRD, and XPS data illustrating the (c) Co 2p, (d) O 1s and (e) Se 3d.

The XPS data are presented in Figure 3c–e. The cobalt system shows peaks corresponding to the Co 2p_1/2_ and Co 2p_3/2_. The Co 2p_3/2_ was further deconvoluted into four peaks with satellite peaks at 790.2 and 786.2 eV. The peak at 782.8 eV is assigned to Co–O,^25^ while the main peak at 781.0 eV is assigned to Co–O, with possible overlap from Co–Se^26^ as metal chalcogenides readily form oxides upon exposure to air, complicating surface analysis, and therefore such species are not easily distinguished in this region.^25,27^ The O 1s spectrum comprises two peaks at 533.3 and 531.6 eV, which are assigned to Se–O and Co–O, respectively.^28^ The Se 3d was deconvoluted into four peaks with a satellite peak at 65.7 eV, and peaks corresponding to Se–O at 62.2 and 60.5 eV^29^ and Co–Se at 55.0 eV. In particular, the peak at 55.0 eV indicates the presence of CoSe_2_.^26,30^ However, the peak at 62.2 eV indicates the presence of selenium oxides, which is consistent with the O 1s, indicating the presence of Se–O at the surface. While this oxidized Se may be due to surface oxidation, it may also point to the electrodeposition of some SeO_2_ at −1.2 V vs Ag/AgCl. Similarly, the Co 2p and O 1s data suggest the presence of cobalt oxides at the surface, consistent with the EDX mapping, which indicates the presence of oxygen. Overall, this analysis verifies that CoSe_2_ was successfully electrodeposited, but it also indicates the codeposition of oxide phases, resulting in cobalt oxides/hydroxides and potentially traces of SeO_2_.

## 4 HER Studies in KOH and H_2_SO_4_ and OER in KOH

### 4.1 HER Studies

The electrodeposited composite comprises not only CoSe_2_ but also cobalt oxide/hydroxides and possibly SeO_2_. To investigate the contributions of these additional components, selenium oxide and cobalt oxide/hydroxide phases were also independently electrodeposited at −1.2 V vs Ag/AgCl for 400 s, and their catalytic activities for HER and OER in 1.0 M KOH were evaluated. The data are presented in Figure S2. This analysis reveals that SeO_2_ exhibits negligible catalytic performance for HER, and consequently has a minimal role in the EDC@GCE. However, it is coelectrodeposited during the formation of the EDC@GCE, and its formation is very difficult to avoid. On the other hand, the electrodeposited cobalt oxide/hydroxide performs well. However, the presence of CoSe_2_ clearly enhances the HER activity, highlighting its critical role in improving the overall electrocatalytic performance.

The HER performance of the electrodeposit in 1.0 M KOH is shown in Figure 4a, where the LSV profiles of the EDC@GCE, the GCE substrate and Pt/C are compared. Clearly, the GCE only acts as a support and does not contribute to the HER activity. The HER activity of the EDC@GCE composite compares well with the benchmark Pt/C electrocatalyst. Indeed, at a current density of *j* = 10 mA cm^–2^, overpotentials (η_10_) of 146 mV and 305 mV were computed for the Pt/C and the electrodeposited EDC@GCE composite, respectively, while the Tafel slope was determined as 69 mV dec^–1^ for the EDC@GCE composite. The EDC@GCE also performed well as a HER electrocatalyst in 0.5 M H_2_SO_4_. The LSVs recorded in 0.5 M H_2_SO_4_ for the EDC@GCE, GCE and Pt/C are shown in Figure 4b. Again, the EDC@GCE composite compares well with Pt/C, with overpotentials of 250 and 99 mV at η_10_, respectively, while the Tafel slope of the composite at 42 mV dec^–1^ is very close to that of 44 mV dec^–1^ reported for highly active and efficient Pt single atoms.^31^ A typical plot highlighting the Tafel region in 0.5 M H_2_SO_4_ is shown in Figure S3a. It is generally recognized that the value of the Tafel slope is closely connected with the HER mechanism. When the rate-determining step is the Volmer reaction, Tafel slopes attain values of approximately 120 mV dec^–1^. Conversely, lower values around 40 and 30 mV dec^–1^ are typically linked to the Heyrovsky step and Tafel reactions, respectively.^32^ The low Tafel slope of 42 mV dec^–1^ in H_2_SO_4_ suggests that the rate-determining reaction at the EDC@GCE is the Heyrovsky reaction, which involves the electrochemical desorption of hydrogen from the EDC@GCE surface.

**Figure 4:**
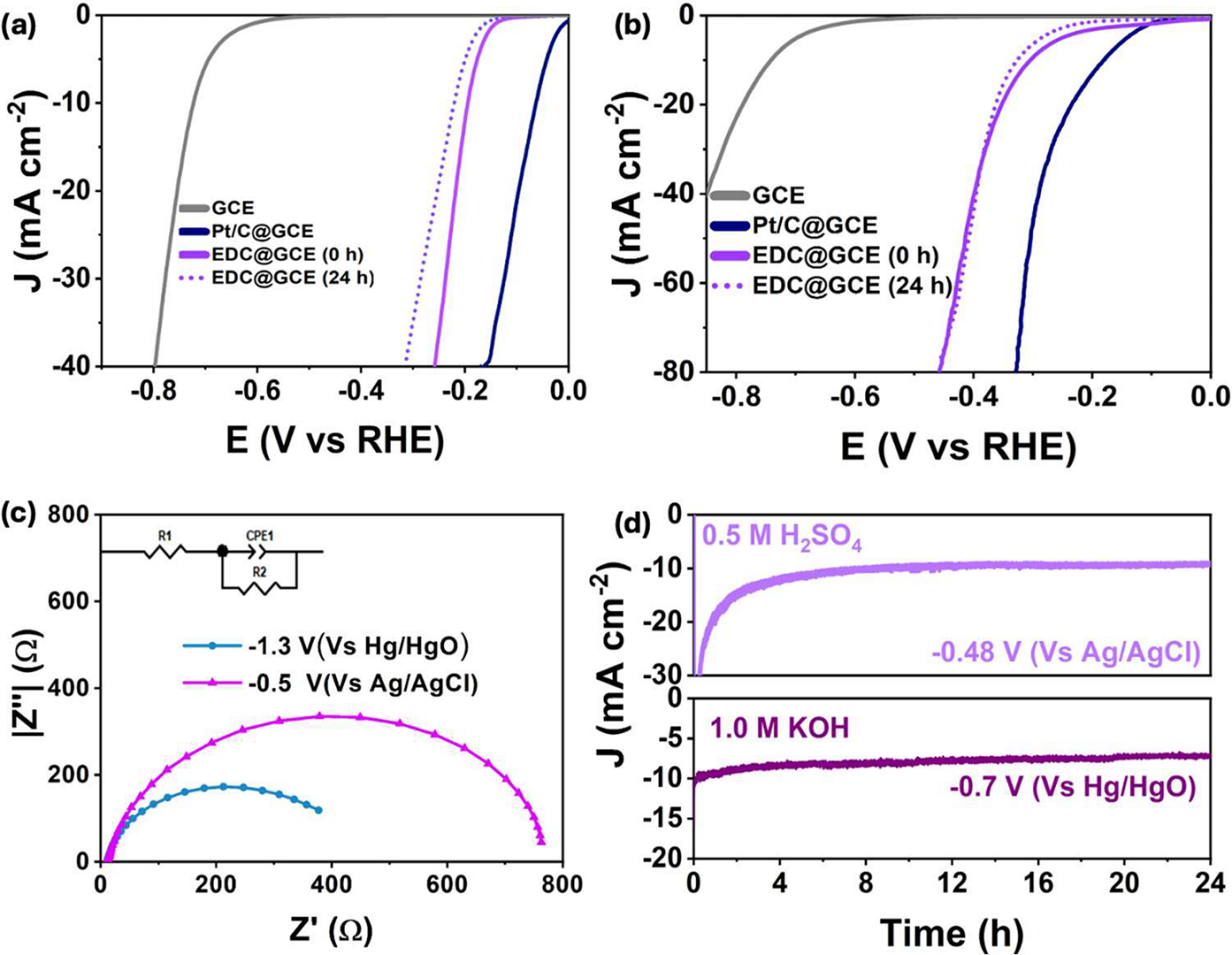
LSVs recorded for Pt/C@GCE, EDC@GCE and GCE in (a) 1.0 M KOH and (b) 0.5 M H_2_SO_4_, (c) impedance spectra recorded in KOH and H_2_SO_4_ and (d) stability over 24 h at a potential corresponding to 10 mA cm^–2^.

In Figure S4, the influence of the composition of the counter electrode (Pt or graphite rods) on the HER activity in both alkaline and acidic media is presented. Chen et al.^20^ have demonstrated that dissolved Pt species can reach concentrations of 0.54 ng·mL^−1^ after immersing Pt plate electrodes in alkaline media for 5 min. The authors concluded that the electrochemical and chemical dissolution of Pt can lead to the deposition of Pt onto the working electrode, resulting in a significant increase in its HER activity. However, as shown in Figure S4, the LSV curves recorded at 5 mV s^–1^ show no evidence of any improvement in the HER activity when a Pt rod counter electrode is employed. This may indicate that Pt is not incorporated at the EDC@GCE surface or that the pure Pt rod is sufficiently stable to limit the release of Pt species into the electrolyte.

The electrochemically active surface area (ECSA) was evaluated using CV data recorded in the non-Faradaic region. Representative CV curves for alkaline (1.0 M KOH) and acidic (0.5 M H_2_SO_4_) media are shown in Figure S5a,b, respectively, exhibiting the characteristic square-like shape associated with capacitance. The capacitive current was recorded and plotted as a function of the scan rate, Figure S5c,d, to determine the *C*_dl_. The ECSA was then computed as *C*_dl_/*C*_s_, where *C*_s_ was set at 0.04 mF cm^–2^. Using this approach, the ECSA was determined to be 89 cm^2^ in 1.0 M KOH and 72 cm^2^ in 0.5 M H_2_SO_4_, compared to a much lower value of 0.07 cm^2^ for the GCE substrate. This notable increase in ECSA is consistent with a significant increase in active sites, which in turn arises from the flower-like morphology observed in Figure 2. The charge transfer resistance was obtained from electrochemical impedance spectroscopy. Typical impedance plots are presented in Figure 4c, recorded in 1.0 M KOH and 0.5 M H_2_SO_4_ in the HER region. These data were fitted to a simple Randles circuit, consisting of a solution resistance in parallel with an RC couple, where *R* corresponds to the charge transfer resistance, *R*_CT_, and *C* represents the capacitance of the system. The *R*_CT_ and *C* values were deduced as 791.4 ± 9.2 Ω cm^2^ and 24.9 ± 7.1 μF cm^–2^ in the 0.5 M H_2_SO_4_ solution, while values of 489.9 ± 5.5 Ω cm^2^ for *R*_CT_ and 210.3 ± 5.7 μF cm^–2^ for *C* were obtained in the 1.0 M KOH solution, suggesting a higher capacitance in the alkaline solution. This is in good agreement with the higher ESCA seen in the KOH solution.

The stability of the electrodeposited composite in both the acidic and alkaline solution is illustrated in Figure 4d. In this case, a fixed potential, corresponding to a current density of 10 mA cm^–2^ in the LSVs, was applied. In both cases, very good stability is achieved over 24 h, with the current remaining stable at approximately 10 mA cm^–2^ over this period. The noise in the current signal is related to hydrogen bubbles, with their continuous formation and detachment giving rise to the slight noise in the measured current. This good stability, in both the acidic and alkaline solutions, appears to be related to the uniform composition of the CoSe_2_/CoO_*x*_, as evident in the mapping study in Figure 2, with the possible transfer of electrons between the oxide and selenide to give a more stable composite, than the individual components. Moreover, the flower-like morphology facilitates the efficient removal of the hydrogen bubbles and the subsequent adsorption of the H^+^ ions.

### 4.2 OER Studies

The EDC@GCE electrodeposited composite was also investigated as an electrocatalyst for the OER. As shown in Figure S2, the SeO_2_ is a very poor OER electrocatalyst. While the electrodeposited cobalt oxide/hydroxide does indeed facilitate the OER, the EDC@GCE is clearly more efficient, again highlighting the important role of the CoSe_2_ in the OER.

Prior to the OER studies, the EDC@GCE was activated by cycling in the KOH and the CVs recorded are shown in Figure 5a. Following approximately 20 cycles, the system reached near steady-state conditions, characterized by a distinct redox reaction that appears to be quasi-reversible with a peak-to-peak separation of 40 mV. This behavior is consistent with the oxidation and reduction of an adsorbed species at the surface of the EDC@GCE. Representative LSV profiles for the EDC@GCE composite, following activation, are presented in Figure 5b, along with the GCE substrate and a Ru oxide rod. The EDC@GCE composite exhibits an onset overpotential of 280 mV for the OER, which compares favorably with that of Ru oxide at 130 mV. Similarly, the overpotential required to give a current density of 10 mA cm^–2^ was 310 mV for the EDC@GCE and 135 mV for the Ru oxide. This indicates that the composite can not only function as an electrocatalyst for HER but also demonstrates good performance in the OER. The Tafel plot is shown in Figure S3c, giving a Tafel slope of 93 mV dec^–1^ for the OER in 1.0 M KOH, which compares well with a number of recent electrocatalysts, including a Tafel slope of 107 mV dec^–1^ for MnCuSe@CNTs,^33^ a slope of 108.5 mV dec^–1^ for (NiCoZnCrFe)­Se,^34^ and a slope of 78.91 mV dec^–1^ for a coassembled CoSe_2_ and MoSe_2_ electrocatalyst.^35^

**Figure 5:**
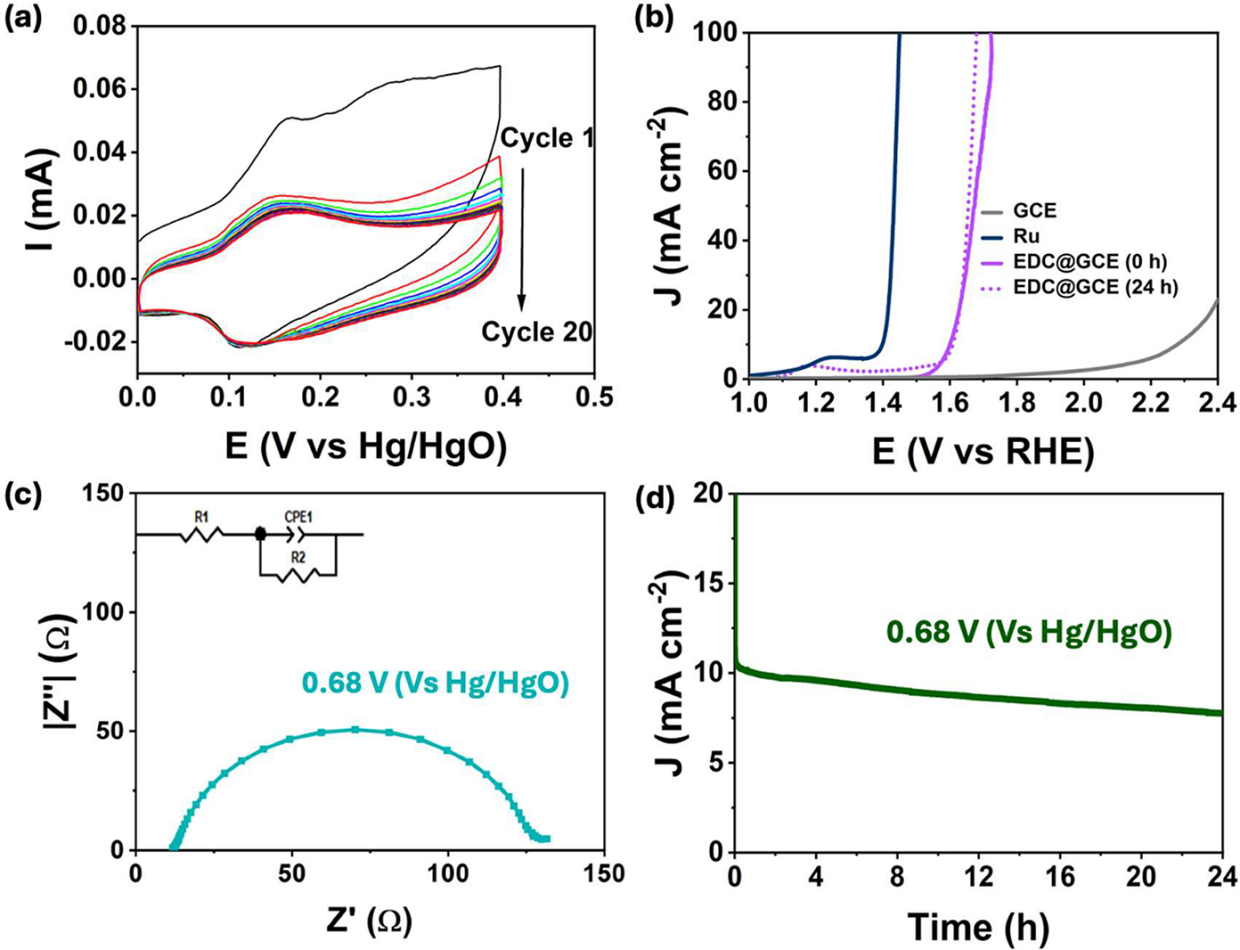
(a) Cycling of the EDC@GCE in 1.0 M KOH, (b) LSV curves recorded for EDC@GCE, Ru rod electrode and GCE substrate, (c) electrochemical impedance spectroscopy recorded in the OER region and (d) stability over a 24 h period, recorded in 1.0 M KOH.

On further inspection of the LSVs it is evident that oxidation waves appear in the vicinity of 1.25 V vs RHE for both the cycled Ru rod and EDC@GCE. The peak associated with the Ru rod is consistent with the formation of ruthenium oxides (RuO_2_), while the oxidation of Co^2+^ to Co^3+^ occurs with an oxidation peak centered at about 1.24 V vs RHE. This is likely to generate cobalt-based oxyhydroxides, such as CoOOH, which are well-known to promote the OER.^36^ Indeed, the low Tafel slope of 93 mV dec^–1^ is consistent with this active CoOOH phase, which can facilitate the multistep kinetics associated with the OER. The impedance profile of the EDC@GCE during the OER is shown in Figure 5c. Again, a semicircle is evident in the Nyquist plot, and upon fitting the data to the Randles cell, the *R*_CT_ was obtained as 117.7 ± 0.7 Ω cm^2^, while the capacitance was computed as 762.1 ± 1.4 μF cm^–2^.

The stability of the EDC@GCE was evaluated at an applied potential corresponding to 10 mA cm^–2^ over 24 h in 1.0 M KOH, Figure 5d. Very good stability was achieved over the 24 h period. Following this period, LSV profiles were recorded and compared to the data obtained with the freshly prepared EDC@GCE electrocatalysts. This comparison is illustrated in Figure 5b, where it is evident that the EDC@GCE exhibits excellent stability. There is no evidence of any loss in the OER activity. Interestingly, an enhancement in OER performance was noted, evidenced by a higher gradient in the current–potential plot following the stability test.

To probe the surface changes at the EDC@GCE during both OER and HER in alkaline and acidic solutions, the XPS data were recorded following the stability experiments at 10 mA cm^–2^. These studies are summarized in Figure 6, where the Se 3d and O 1s XPS spectra are shown following stability testing under HER conditions (in both 1.0 M KOH and 0.5 M H_2_SO_4_), and OER conditions (in 1.0 M KOH). After HER chronoamperometry in alkaline media, Figure 6a, the Se 3d spectrum exhibits a more dominant Se–O peak at 61.7 eV with a reduced signal from the Co–Se peak at 57.6 eV, suggesting increased surface oxidation in this alkaline medium. The corresponding O 1s spectrum is shown in Figure 6b and reveals the presence of Co–O bonding, indicating an oxide/hydroxide-rich surface composition. In contrast, for the post-HER in the acidic medium, Figure 6c,d, the Se–O and Co–Se are retained along with the Se–Se bonding, suggesting a greater presence of selenide species at the surface in the acidic medium. Notably, the O 1s region is dominated by Co–O and Se–O signals, but with less oxide contributions than in the KOH solution. For OER conditions, Figure 6e, the O 1s spectrum exhibits pronounced Co–O peaks associated with cobalt (Co^2+^ and Co^3+^) oxide and hydroxide phases after prolonged OER conditions. The Se 3d signals were below reliable detection limits, indicating significant Se loss or surface passivation after prolonged anodic bias, which is consistent with reports of selenide-oxide conversion under both HER and OER conditions in alkaline medium.^37,38^ However, as shown in the EDX spectrum, Figure S6, the bulk composition retains the selenide, indicating that the conversion occurs solely at the surface exposed to the electrolyte. Overall, these spectra confirm that the surface proceeds towards conversion to cobalt oxides/hydroxides, such as the active Co^3+^ (Co­(OOH)) phase over time, with the Co–Se species being more stable in acid than alkaline media.

**Figure 6:**
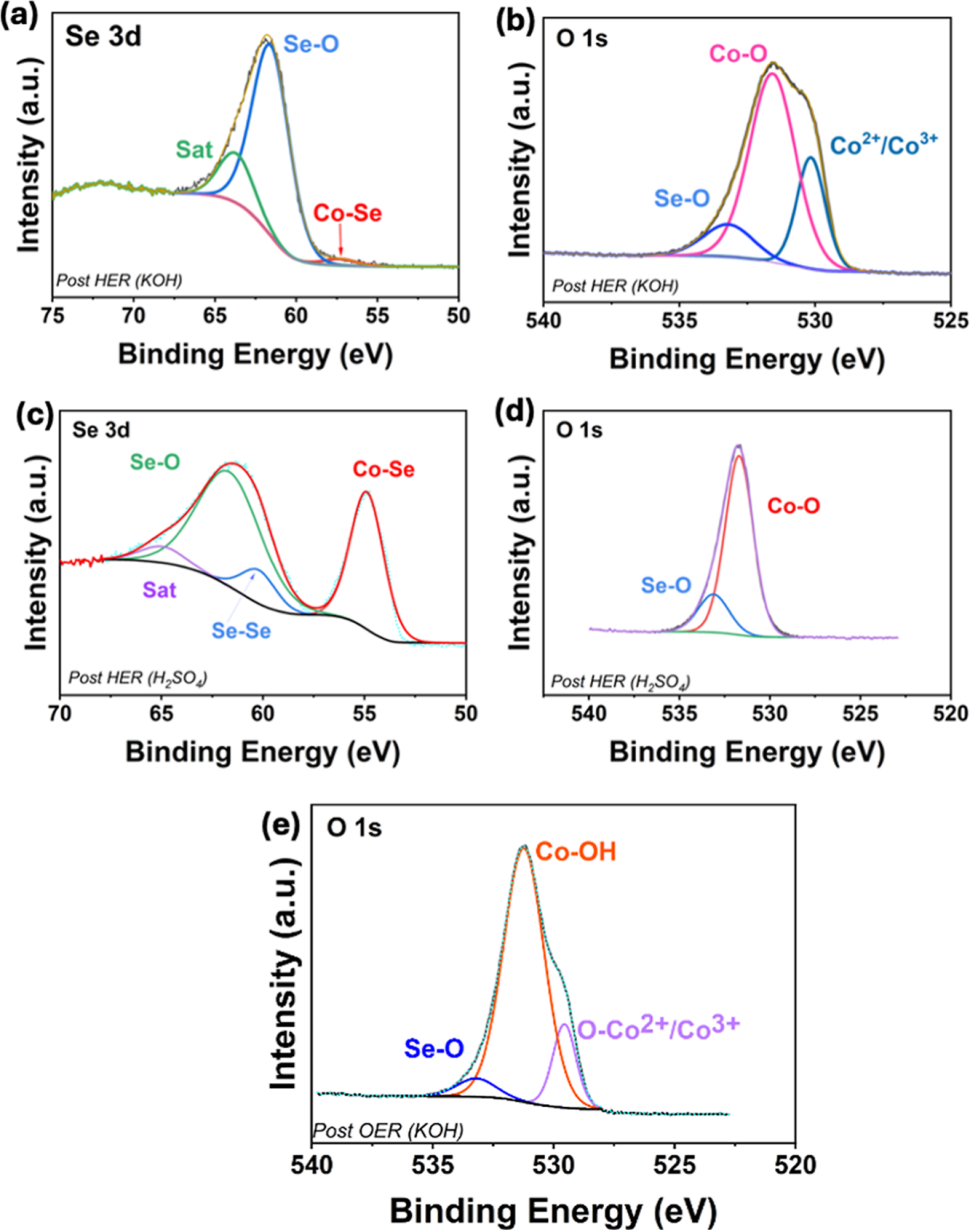
Post stability XPS analysis (a) Se 3d and (b) O 1s following HER stability in KOH, (c) Se 3d and (d) O 1s following HER stability in H_2_SO_4_, and (e) O 1s following OER stability in KOH.

It has been shown that iron impurities in commercial KOH can be readily incorporated within Ni-based hydroxide films. Indeed, iron concentrations as low as 1 ppm can alter significantly the activity of Ni­(OH)_2_–based electrocatalysts.^21,39^ Therefore, the somewhat higher OER activity observed in Figure 5c was further investigated by considering the role of trace amounts of iron in the KOH solution. The KOH was purified, as indicated in the experimental section, and the OER activity was recorded in this pure KOH solution. This comparison is illustrated in Figure S7, where it is evident that residual iron in the solution phase can indeed enhance the OER activity. In the case of Ni-based oxide electrocatalysts, it has been suggested that the incorporation of iron can enhance the electrical conductivity of the electrocatalyst.^39^ Alternatively, an Fe-induced partial-charge transfer mechanism has been proposed,^21^ whereby the incorporated iron activates the Ni centers within the electrocatalysts. A similar process appears to occur with the EDC@GCE electrocatalyst, resulting in the apparent increase in OER activity following the 24 h stability test. Indeed, the peaks observed in Figure 5a, which are also shown in the inset in Figure S7, may be associated with the interconversion between Fe^2+^ and Fe^3+^, indicating the incorporation of iron at the surface of the EDC@GCE electrocatalysts. Moreover, the peaks at about 1.05 V vs RHE in Figures 5b and S7, are consistent with the oxidation of Fe^2+^ to Fe^3+^, while the peak observed at the higher potential of about 1.27 V is consistent with the oxidation of Co^2+^ to Co^3+^. This analysis indicates that the EDC@GCE is susceptible to the incorporation of trace amounts of iron from the KOH solution.

## 5 Conclusions

A simple and rapid electrodeposition process, requiring 400 s, was employed to form a flower-like hierarchical Co-based oxide/selenide composite with a high surface area and accessible leaf-like sheets with active edges. These structural features enhance both the HER and OER activity. The composite has good stability over a 24 h period and good overpotentials of 310 mV for the OER in 1.0 M KOH, and 306 mV and 250 mV for the HER in 1.0 M KOH and 0.5 M H_2_SO_4_, respectively, to deliver a current density of 10 mA cm^–2^. The hierarchical structure provides a high surface area that contributes to the efficient OER activity in alkaline media and the good HER performance in both acidic and alkaline media. Nevertheless, a key advantage of this electrocatalyst is the very short electrodeposition period required for its deposition. Although a simple GCE electrode was used in this study, the electrodeposition process is suitable for the deposition of the composite on other conducting supports.

## Supplementary Material



## Notes

Overpotential for HER as a function of electrodeposition period, Figure S1; LSVs for the individual components in the HER, Figure S2; Tafel plots in acidic and alkaline media, Figure S3; LSVs recorded for HER using a Pt counter electrode, Figure S4; CVs recorded in the capacitive region, Figure S5; EDX following stability study, Figure S6 and LSVs recorded in purified KOH, Figure S7 (PDF)
