# Supplementary material for: Hydrogen Evolution-Directed Electrodeposition of a Cobalt Selenide/Cobalt Oxide Electrocatalyst for the Hydrogen and Oxygen Evolution Reactions
Source: ACS Appl Energy Mater. 2025 Aug 6;8(16):12405–14. doi: 10.1021/acsaem.5c02096 (PMC12381822; doi:10.1021/acsaem.5c02096)
Supplement: Supplementary file 1 [file ae5c02096_si_001.pdf]

Hydrogen evolution-directed electrodeposition of a cobalt selenide/cobalt oxide electrocatalyst for the hydrogen and oxygen evolution reactions

Gillian Collins[1], Daniele Alves[1], Tara Barwa[1], Karthik Raj[1], Ramaraj Sukanya [1], and Carmel B. Breslin[*1,2]

[1] Department of Chemistry, Maynooth University, Maynooth, Co. Kildare, Ireland

[2] Kathleen Lonsdale Institute, Maynooth University, Maynooth, Co. Kildare, Ireland

*Corresponding author: Carmel.Breslin@mu.ie

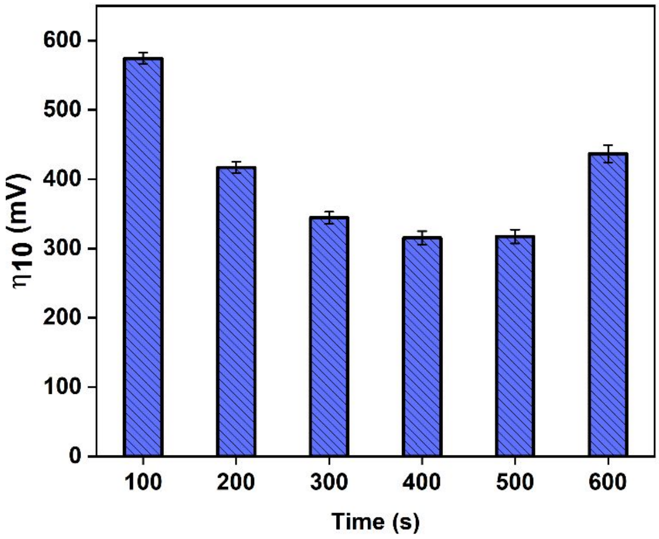

Figure S1: Overpotential at 10 mA cm^{-2} measured for the HER in 1.0 M KOH as a function of the electrodeposition period at –1.2 V vs Ag/AgCl in 20 mM $(CH_3COO)_2Co\cdot 4H_2O$, 5 mM Na_2SeO_3, and 0.1 M KCl.

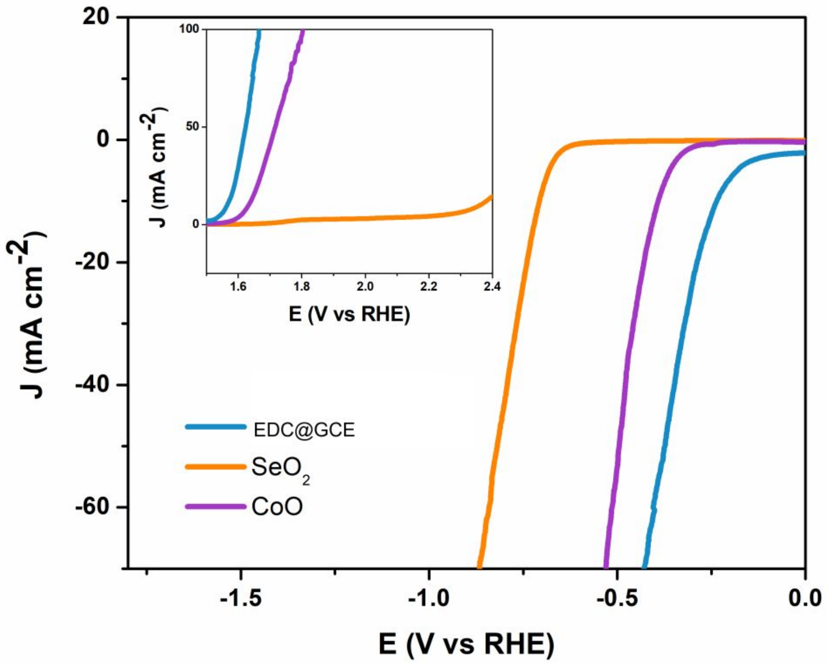

Figure S2: LSV recorded for the EDC@GCE, electrodeposited SeO_2 and electrodeposited cobalt oxide in 1.0 M KOH at 10 mV s^{-1} for the HER with the inset showing the corresponding data for the OER in 1.0 M KOH.

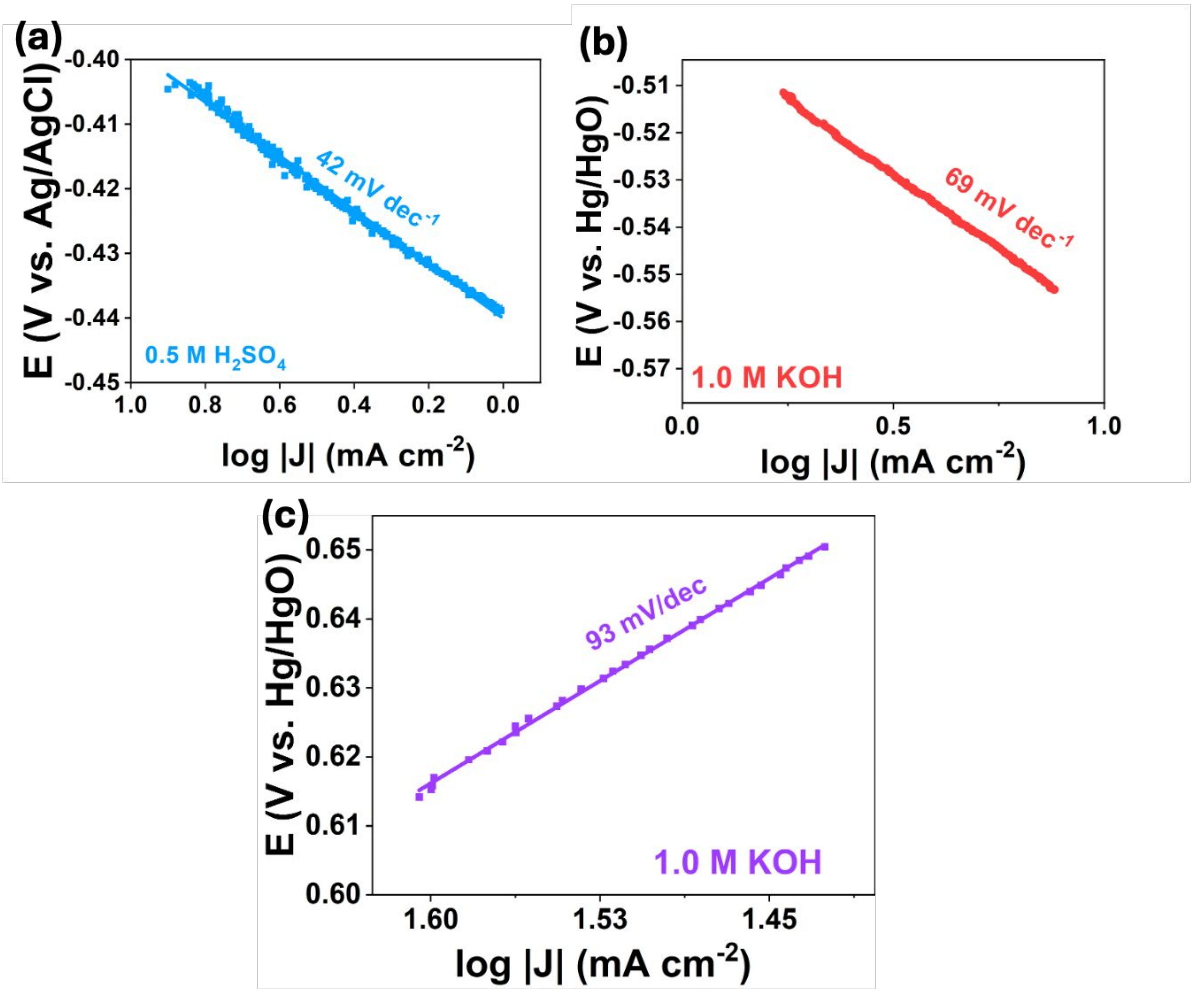

Figure S3: Tafel plots recorded in (a) 0.5 M H_2SO_4 for the HER, (b) in 1.0 M KOH for the HER and (c) in 1.0 M KOH for the OER.

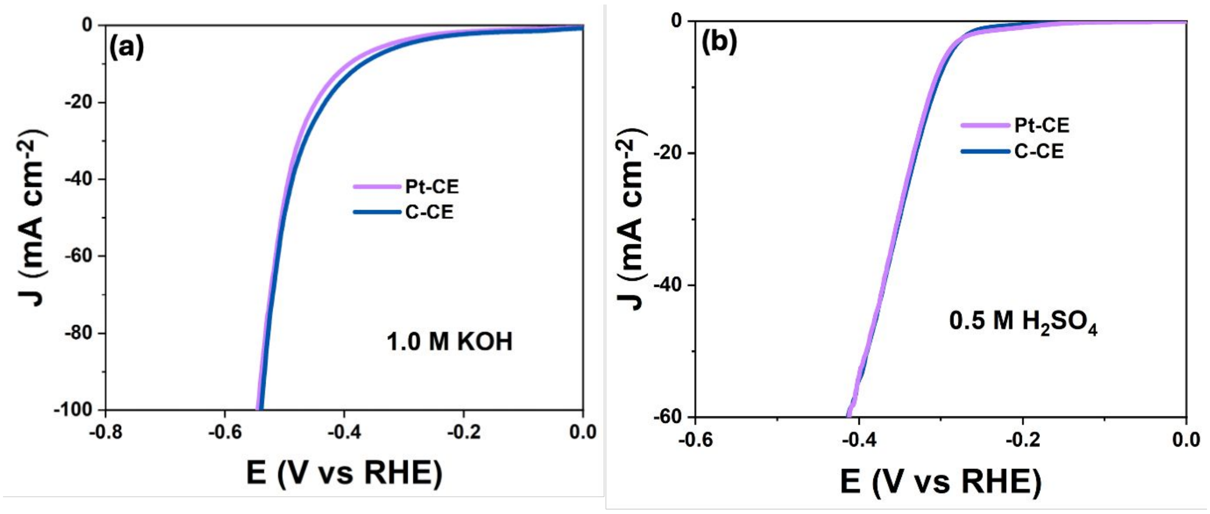

Figure S4: LSV curves recorded at 10 mV s^{-1} using a Pt counter electrode (Pt-CE) and a carbon counter electrode (C-CE) in (a) 1.0 M KOH and (b) 0.5 M H_2SO_4.

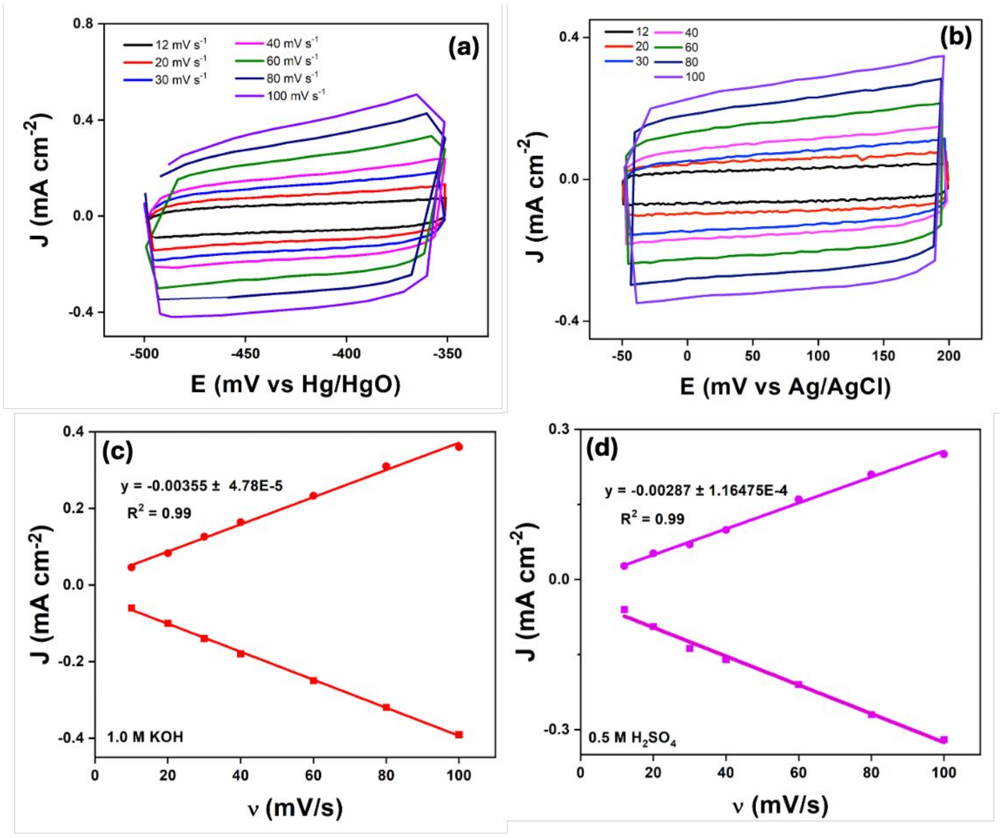

Figure S5: CVs recorded in the capacitive region in **(a)** 1.0 M KOH and **(b)** 0.5 M H_2SO_4 and corresponding current as a function of scan rate for data recorded in **(c)** 1.0 M KOH and **(d)** 0.5 M H_2SO_4.

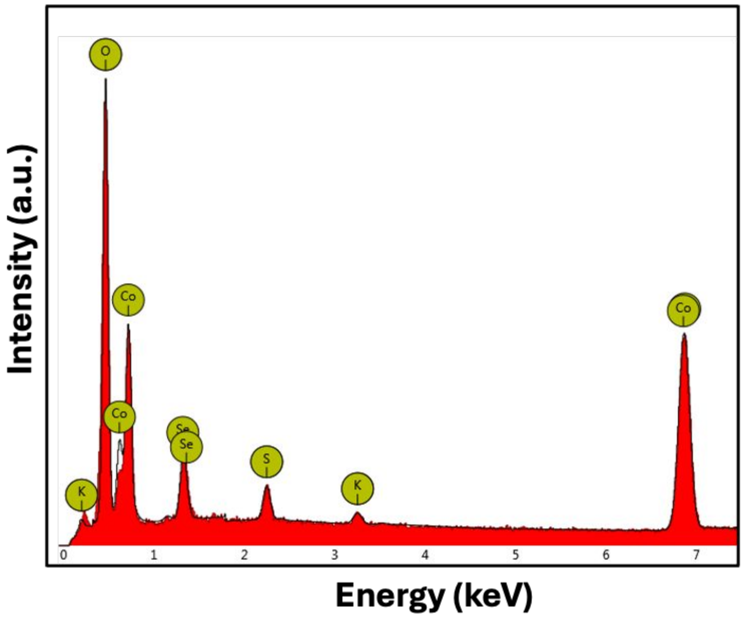

Figure S6: EDX recorded following the stability studies, indicating the presence of bulk Se and Co and O.

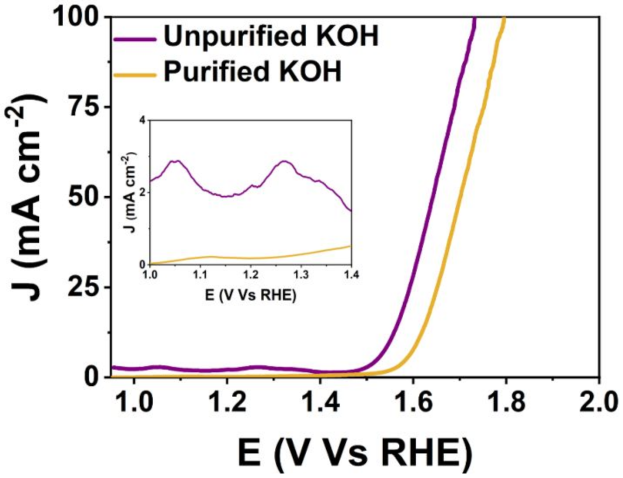

Figure S7: LSV curves recorded in 1.0 M KOH at 10 mV s^{-1} in purified KOH and commercial KOH.
